# Bactericidal and virucidal efficacies of food additive grade calcium hydroxide under various concentrations, organic material conditions, exposure duration, and its stability

**DOI:** 10.14202/vetworld.2019.1383-1389

**Published:** 2019-09

**Authors:** Sakchai Ruenphet, Kornkamon Paditporn, Darsaniya Punyadarsaniya, Tippawan Jantafong, Kazuaki Takehara

**Affiliations:** 1 Department of Immunology and Virology, Faculty of Veterinary Medicine, Mahanakorn University of Technology, Bangkok, Thailand; 2 Laboratory of Animal Health, Department of Veterinary Medicine, Faculty of Agriculture, Tokyo University of Agriculture and Technology, Tokyo, Japan

**Keywords:** bactericidal, biosecurity, food additive grade calcium hydroxide, virucidal

## Abstract

**Aim:**

This study aimed to evaluate the bactericidal and virucidal activity of food additive grade calcium hydroxide (FdCa(OH)_2_) under various concentrations, organic material conditions, and exposure duration including its stability.

**Materials and Methods:**

The FdCa(OH)_2_ powder as well as the 0.17% and 3% solutions were evaluated for bacteria and virus inactivating efficacies against *Salmonella* infantis (SI), *Escherichia coli*, Newcastle disease virus (NDV), and avian influenza virus (AIV), in the absence or presence of organic materials. In addition, the stability of FdCa(OH)_2_, was also examined using wet-dry conditions and under sunlight.

**Results:**

The FdCa(OH)_2_ powder could inactivate both NDV and AIV in the absence and presence of organic materials within a 3 min exposure period. The bactericidal efficacy using solution form revealed that 0.17% and 3% of FdCa(OH)_2_ could inactivate SI in the absence and presence of organic materials within 3 min of exposure. However, 3% of FdCa(OH)_2_ inactivated *E. coli* both with and without organic materials within 3min, while 0.17% required 5 min to be efficacious. The virucidal efficacy also showed that 0.17% FdCa(OH)_2_ could inactivate NDV in the absence and presence of organic materials within 10 min and 30 min, respectively. However, AIV inactivation was achieved within 30 sec under all conditions. In addition, under wet and dry conditions, FdCa(OH)_2_ powder demonstrated high efficacy when re-suspended at least 16 times for NDV and 7 times for AIV. Simultaneously, the FdCa(OH)_2_ powder retained its efficacy under the sunlight during up to 4 months for NDV and at least 6 months for AIV.

**Conclusion:**

The present study indicates that FdCa(OH)_2_ powder and solutions could inactivate SI, *E. coli*, NDV, and AIV while retaining good stability under challenging environmental conditions. Finally, the FdCa(OH)_2_ is safe for consumers because it is of food additive grade and can be useful as an alternative disinfectant, especially for biosecurity enhancement on and around poultry farms.

## Introduction

Food-borne diseases causing *Escherichia coli* and *Salmonella* spp. are major public health concerns worldwide, especially in the United States, Japan, and Thailand. A variety of foods from animals, such as eggs, meat, and milk, have been implicated as vehicles of one or more of these pathogens in outbreaks of food-borne illness [1]. The U.S. Department of Agriculture, Food Safety, and Inspection Service recommends the Pathogen Reduction Program for antimicrobial treatments, which is a method for reducing or inactivating pathogenic bacteria in foods [2]. The hazard analysis and critical control point (HACCP) has been required for food safety and was introduced at standard farms in Japan. This HACCP will be used for the establishment of critical control points in restaurants, homes, and other foodservice units, including the Olympic games of 2020 in Japan [3]. For animal farms, appropriate HACCP work is necessary to enhance biosecurity, especially in terms of appropriate disinfectants. Several respiratory viruses such as Newcastle disease and bird flu causing Newcastle disease virus (NDV) and avian influenza virus (AIV), have a strong negative impact on the economy of the poultry industry. In general, infected birds are excreted a lot of viruses from the respiratory and gastrointestinal system such as nasal discharge, feces, and leading to transmission and contamination in and around animal farms including human and environment. Hence, instruments of disease control are cleaning and disinfectant applying; however, the efficacy depends on the property of disinfectant and environment condition in animal farm. Many disinfectants are available commercially, and it is important to ensure that the disinfectant being used is effective against various pathogens. The modern commercial farms and their environment can provide an appropriate medium for bacteria and virus propagation, while high animal population and short downtimes contribute to high disease incidence [4]. The effective management and husbandry practices are the all-in all-out system [5] and regular cleaning and disinfection, especially before introducing a new flock or herd to the barns [6]. These applications are effective methods to reduce the presence of any bacteria and viruses.

The appropriate disinfectants are critical in establishing a successful sanitation program. Nevertheless, since not all disinfectants are effective against the major pathogens, different families of disinfectants that target specific microorganism should be considered. For instance, several bacteria and viruses are sensitive to phenols, while most bacteria are more sensitive to quaternary ammonium, iodophor, peracetic acid, glutaraldehyde, and cresols [7]. Therefore, there is no one disinfectant reported in the literature that would be efficacious against a wide spectrum of etiological agents of economically impacting diseases in animal farms. Moreover, special care should be taken in the application of the disinfectant, as it should be safe for both animals and humans. In addition, the hardness of water, correct dilutions, contact duration, and the presence of organic material should also be taken into consideration. Several alkaline disinfectants were reported such as ceramic powder [8], nano-sized scallop shell powder [9], scallop shell powder [10], and calcinated eggshell [11]. Nowadays, several forms of calcium hydroxide (Ca(OH)_2_) such as powder form and 0.17% of Ca(OH)_2_ solution have been shown to exhibit bactericidal efficacies [12,13] and virucidal efficacies [14,15].

This study aimed to evaluate the efficacy of food additive grade calcium hydroxide (FdCa(OH)_2_) powder, using powder and solution forms against *Salmonella* infantis (SI), *E. coli*, NDV, and AIV, in the absence or presence of organic materials, including the stability of FdCa(OH)_2_ while applied for biosecurity in and around poultry farms.

## Materials and Methods

### Ethical approval

Ethical approval is not needed to pursue this type of study.

### Samples and sample preparation

The FdCa(OH)_2_ powder (Fine Co., Ltd., Japan) was used for the present study. The suspension of FdCa(OH)_2_ was prepared as 0.17% or 3% (w/v) using distilled water and then centrifuged at 3000× g for 10 min, the supernatants were used as solution sample in the absence of organic material. The presence of organic material was simulated by adding fetal bovine serum (FBS) as 5% FBS to the solution sample.

### Pathogens, cells, and media

*E. coli* and SI provided by Asst. Prof. Dr. Walaiporn Tonpitak (Microbiology section, Mahanakorn Veterinary Diagnostic Center, Veterinary Medicine Faculty, Mahanakorn University of Technology [MUT], Thailand), were used for the present study. These bacteria were sub-cultured onto deoxycholate hydrogen sulfide lactose (DHL) agar and then incubated at 37°C overnight. The bacterial colony was picked up and cultivated in Luria-Bertani medium (1% Bacto Tryptone, 0.5% Bacto Yeast Extract, and 1%NaCl, pH 7.4) and titrated on DHL agar [11]. Organic materials were removed from bacteria cultures by centrifugation at 1750× g for 10 min before testing. In addition, a virulent NDV, namely, NDV/chicken/Asean Country/2013 [16] and a low pathogenic AIV, namely, A/duck/Asean Country/2004 H9N2 that was provided by Prof. Thaweesak Songserm (Department of Pathology, Faculty of Veterinary Medicine, Kasetsart University), were propagated in 9-day-old chicken embryonic eggs. After allantoic fluid harvesting at 3-day post-inoculation, stock viruses were aliquoted and kept at −80°C until testing. The chicken embryo fibroblasts (CEF) and Madin-Darby canine kidney (MDCK) cells were used for NDV and AIV titration, respectively.

### Blocking solution preparation

The activity of FdCa(OH)_2_ solution was stopped with blocking solution. In the present study, this solution was prepared from 1M Tris-HCl pH 7.2 as described [12].

### Antimicrobial properties of FdCa(OH)_2_ powder

To determine virus inactivation properties of the FdCa(OH)_2_ powder, 200 mg of FdCa(OH)_2_ powder was mixed with 100 µl of NDV or AIV in the absence of organic materials. The efficacy of the same powder was then evaluated in the presence of organic material simulated using FBS as virus containing 33% of organic materials. Briefly, 100 µl of each virus was mixed with 50 µl of FBS, and then added all mixed to 300 mg of FdCa(OH)_2_ powder. After 3 min incubation at room temperature, the viruses were recovered using phosphate-buffered saline (PBS) and titrated onto CEF or MDCK cells, respectively [11].

### Antimicrobial properties of FdCa(OH)_2_ solution

Four hundred microliters of each FdCa(OH)_2_ solution sample were mixed with 100 µl of each pathogen, then incubated at room temperature for 5 s, 30 s, 1 min, 3 min, 5 min, 10 min, 30 min, or 1 h. After that, the solution mixture was neutralized with 500 µl of blocking solution, and then titrated onto MDCK cells and CEF for AIV and NDV, respectively, or onto DHL agar Petri dish for both bacteria. To simulate the presence of organic materials, 500 µl of FBS was added to 10 ml of each FdCa(OH)_2_ solution sample to represent 5% organic material, before testing. To confirm the neutralizing efficacy of Tris-HCl, it was added into each solution sample before adding virus or bacteria, namely, at 0 s. Each treatment was conducted in triplicates, and the titers were expressed as means with standard error (SE).

### Stability under sunlight and wet-dry condition

The stability of FdCa(OH)_2_ powder stored under harsh conditions was also evaluated for its virucidal activity using NDV and AIV. A quantity of 3 g of FdCa(OH)_2_ powder in a 90-mm Petri dish was kept under sunlight for 6 months. Another batch of FdCa(OH)_2_ powder was used for making suspensions in 10 ml of distilled water, and the dish was kept until completely dried in 37°C incubator. Re-suspension and drying were repeated up to 16 times or until virucidal efficacy losing [9].

### Virus and bacteria titration and calculation

Each *E. coli* and SI treatment was diluted as 10-fold serial dilution using PBS and inoculated onto DHL agar for bacterial titration. All inoculated Petri dishes were incubated at 37°C incubator, and the number of colonies was recorded at 24-h post-inoculation. The bacteria titer was calculated in colony-forming units/ml. In addition, NDV and AIV treated samples were diluted as 10-fold serial dilution using Eagle’s minimum essential medium (MEM, Nissui Pharmaceutical Co., Ltd., Tokyo, Japan) containing 0.3% of tryptose phosphate broth, penicillin 100 units/ml, streptomycin 100 μg/ml, amphotericin B 0.5 μg/ml, and 4 mM L-glutamine and inoculated onto CEF or MDCK, respectively. However, before inoculation, trypsin (Trypsin, Sigma, St. Louis, MO, USA), at the final concentration of 0.2 µg/ml was added to MEM. All of inoculated tissue culture plates were incubated at 37°C in 5% CO_2_ incubator and observed for cytopathic effect (CPE) twice a day for 3 days. At the end of the incubation period, the hemagglutinin activity of the culturing supernatant was detected using 0.5% chicken red blood cells. Finally, the 50% tissue culture infective dose/ml was determined by Behrens and Kärber’s method [9].

### Inactivation analysis

The reduction factor (RF) was used for determining the bacteria or virus inactivation. The RF was calculated using the following equation RF = t_pc_−t_a_; where t_pc_ is the titer converted into an index in log_10_ of the positive control, and t_a_ is the converted titer an index in log_10_ of the recovered bacteria or virus from the treated sample. Bacteria or virus inactivation was considered effective when RF was greater than or equal to 3 log_10_ [17-19].

### Statistical analysis

In the present study, the RF was analyzed independently and shown as mean ± SE. The one-way analysis of variance *post* *hoc* test (SPSS, Armonk, NY, USA) was performed to determine the statistical significance of differences in FdCa(OH)_2_ efficacy between the positive control and also among the treatment group. A significant difference was noticed while the associated p<0.05.

## Results

### Virucidal efficacy using the powder form

The inactivating efficacy of the FdCa(OH)_2_ powder is shown in Table-1. FdCa(OH)_2_ powder could inactivate both NDV and AIV in the absence of organic material, with the RF being >5.17±0.88 and >3.75±0.25, respectively, and in the presence of organic materials being 4.58±1.28 and >4.00±0.25, respectively (Table-1).

**Table 1:** The results are presented as log_10_ TCID_50_/ml (mean±SE) of Newcastle disease virus and avian influenza virus inactivating efficacy, using food additive grade calcium hydroxide powder, in the absence or presence of organic materials.

Organic materials	Newcastle disease virus			Avian influenza virus		
	
	Treated	Control	RF^a^	Treated	Control	RF
0%	<2.50±0.00	7.67±0.88	>5.17±0.88*	<2.50±0.00	6.25±0.25	>3.75±0.25*
33%	<2.50±0.00	7.08±1.28	>4.58±1.28*	<2.50±0.00	6.50±0.25	>4.00±0.25*

a RF=Reduction factor,

* Inactivation regarded as effective when RF was ≥3 log_10_. TCID_50_=50% tissue culture infective dose

### Bactericidal efficacy using solution form

Table-2 shows the bactericidal efficacy of FdCa(OH)_2_ solution against SI and *E. coli* in the absence or presence of organic materials. Both 0.17% and 3% of FdCa(OH)_2_ solution could inactivate SI in the absence and presence of organic materials within 3 min; nevertheless, only 3% of FdCa(OH)_2_ solution could inactivate *E. coli* – regardless of the presence or absence of organic materials – within 3 min. However, the 0.17% solution requires 5 min to inactivate the *E. coli*.

**Table 2:** The results are presented as log_10_ CFU/ml (mean±SE) of *Salmonella* infantis and *E. coli* inactivating efficacy, using 0.17% and 3% of food additive grade calcium hydroxide solution, in the absence or presence of organic materials.

Conditions	*Salmonella* infantis				*E. coli*			
	
	0.17%		3%		0.17%		3%	
			
	0% FBS	5% FBS^a^	0% FBS	5% FBS	0% FBS	5% FBS	0% FBS	5% FBS
tpc^b^	8.65±0.19	8.65±0.19	8.65±0.19	8.65±0.19	9.10±0.19	9.10±0.19	9.10±0.19	9.10±0.19
0 s^c^	8.85±0.51	8.70±0.17	8.67±0.20	8.69±0.14	9.09±0.45	8.84±0.25	8.94±0.31	8.75±0.32
1 min^d^	6.73±0.66	6.66±0.52	6.38±0.38	6.30±0.48	7.60±0.00	7.60±0.00	7.60±0.00	7.44±0.29
3 min	2.70±0.17*	3.25±0.75*	3.38±1.34*	3.51±1.58*	6.57±0.49	6.96±0.36	4.51±0.27*	5.31±0.41*
5 min	2.60±0.00*	2.70±0.17*	2.60±0.00*	2.60±0.00*	3.81±1.05*	4.24±1.44*	2.60±0.00*	2.60±0.00*

a Fetal bovine serum was added to FdCa (OH)_2_ solution so as to represent 5% organic materials of total volume.

b The titer converted into an index in log_10_ of bacteria control.

c The titer in log_10_ that added blocking solution before *Salmonella* infantis or *E. coli*.

d The titer converted into an index in log_10_ of the recovered bacteria after indicated time of treatment, such as 1 min, 3 min, and 5 min.

* Inactivation regarded as effective when RF was ≥3. *E. coli*=*Escherichia coli*, RF=Reduction factor, CFU=Colony-forming units, tpc=Titer of bacteria/virus control, FBS=Fetal bovine serum, FdCa (OH)_2_=Food additive grade calcium hydroxide

### Virucidal efficacy using solution form

The virucidal efficacy of FdCa(OH)_2_ solution against NDV and AIV is depicted in Table-3. At 0.17% of FdCa(OH)_2_, solution could inactivate NDV in the absence and presence of organic material within 10 min and 30 min, respectively. Conversely, AIV was inactivated within 30 sec, regardless of the presence and absence of organic materials.

**Table 3:** The results are presented as log_10_ TCID_50_/ml (mean±SE) of Newcastle disease virus and avian influenza virus inactivating efficacy, using 0.17% and 3% of food additive grade calcium hydroxide solution, in the absence or presence of organic materials.

Conditions	Newcastle disease virus				Avian influenza virus			
	
	0.17%		3%		0.17%		3%	
			
	0% FBS	5% FBS^a^	0% FBS	5% FBS	0% FBS	5% FBS	0% FBS	5% FBS
tpc^b^	8.13±0.43	8.25±0.47	8.13±0.43	8.13±0.43	6.50±0.35	6.50±0.35	6.31±0.31	6.31±0.31
0 s^c^	8.17±0.38	8.25±0.66	8.08±0.29	8.00±0.50	6.25±0.43	6.50±0.50	6.19±0.72	6.50±0.58
5 s^d^	NT^e^	NT	NT	NT	3.92±0.76	4.00±0.25	3.33±1.04	3.33±1.44
30 s	NT	NT	NT	NT	2.92±0.52*	2.50±0.00*	2.92±0.29*	2.50±0.00*
1 min	NT	NT	NT	NT	NT	NT	NT	NT
5 min	5.63±0.18	6.13±0.53	5.25±0.00	5.38±0.18	NT	NT	NT	NT
10 min	4.67±0.14*	5.83±0.76	4.75±0.25*	5.00±0.66*	NT	NT	NT	NT
30 min	4.13±0.88*	5.08±0.29*	4.25±0.71*	4.25±1.06*	NT	NT	NT	NT
1 h	NT	4.75±0.00*	NT	NT	NT	NT	NT	NT

a Fetal bovine serum was added to FdCa (OH)_2_ solution so as to represent 5% organic materials of total volume.

b The titer converted into an index in log_10_ of virus control.

c The titer in log_10_ while adding blocking solution before Newcastle disease virus or avian influenza virus.

d The titer converted into an index in log_10_ of the recovered virus after indicated time of treatment, such as 5 s, 30 s, 1 min, 5 min, 10 min, 15 min, 30 min, and 1 h.

e NT=Not tested.

* Inactivation regarded as effective when RF was ≥3. tpc=Titer of bacteria/virus control, FBS=Fetal bovine serum, FdCa (OH)_2_=Food additive grade calcium hydroxide

### Stability of FdCa(OH)_2_

Results depicting the outcome of the evaluation of FdCa(OH)_2_ powder kept under conditions simulating a harsh environment such as wet and dry conditions and under sunlight, as shown in Tables-4 and 5. Even under wet and dry conditions, Ca(OH)_2_ powder demonstrated high efficacy when re-suspended ≥16 times for NDV and 7 times for AIV (Table-4). The viral titer was reduced to below the detection limit using the FdCa(OH)_2_ powder that was re-suspended 8 times for NDV, and 5 times for AIV. In addition, the FdCa(OH)_2_ powder was also shown to be able to retain its efficacy under sunlight for up to 4 months for NDV and at least 6 months for AIV (Table-5).

**Table 4:** Efficacy of food additive calcium grade hydroxide powder for inactivating Newcastle disease virus and avian influenza virus under wet and dry conditions at consecutive re-suspension times, with a 3-min incubation period.

Number of resuspension	Newcastle disease virus			Avian influenza virus		
	
	Treated	Control	RF^a^	Treated	Control	RF
1	<2.50	8.25	>5.75*	<2.50	6.00	>3.50*
2	<2.50	8.25	>5.75*	<2.50	6.00	>3.50*
3	<2.50	8.25	>5.75*	<2.50	6.00	>3.50*
4	<2.50	8.25	>5.75*	<2.50	6.00	>3.50*
5	<2.50	8.25	>5.75*	<2.50	6.00	>3.50*
6	<2.50	8.25	>5.75*	3.50	6.50	3.00*
7	<2.50	8.25	>5.75*	3.25	6.25	3.00*
8	<2.50	8.25	>5.75*	3.50	6.25	2.75
9	5.25	8.75	3.50*	3.50	6.25	2.75
10	5.00	8.75	3.75*	4.00	6.25	2.25
11	5.25	8.75	3.50*	NT^b^	NT	NT
12	5.00	8.75	3.75*	NT	NT	NT
13	5.25	8.75	3.50*	NT	NT	NT
14	5.50	8.75	3.25*	NT	NT	NT
15	5.50	8.75	3.25*	NT	NT	NT
16	5.25	8.75	3.50*	NT	NT	NT

a RF=Reduction factor,

b NT=Not tested.

* Inactivation regarded as effective when RF was ≥3

**Table 5:** Efficacy of food additive grade calcium hydroxide powder at consecutive time points for inactivating Newcastle disease virus and avian influenza virus under sunlight, during a 3-min incubation period.

Month	Newcastle disease virus			Avian influenza virus		
	
	Treated	Control	RF^a^	Treated	Control	RF
1	<2.50	8.75	>6.25*	<2.50	6.25	>3.75*
2	<2.50	8.75	>6.25*	<2.50	6.25	>3.75*
3	<2.50	8.75	>6.25*	<2.50	6.25	>3.75*
4	<2.50	7.50	>5.00*	<2.50	6.25	>3.75*
5	7.25	7.50	0.25	<2.50	6.25	>3.75*
6	6.25	7.50	1.25	<2.50	6.25	>3.75*

a RF=Reduction factor,

* Inactivation regarded as effective when RF was ≥3

## Discussion

Titer of bacteria/virus control and 0 s did not show marked inactivation difference; these results indicated that the efficacy of blocking solution could neutralize the inactivation activity of FdCa(OH)_2_. This blocking solution was used as an instrument for the determination of contact time in the present study. Sonthipet *et al*. [20] also used 1 M Tris-HCl as blocking solution for exposure time determination to bactericidal and virucidal efficacies of potassium monopersulfate. The present study suggests that the inactivating mechanism of FdCa(OH)_2_ might be associated with high alkalinity.

This FdCa(OH)_2_ is made from Japanese marine limestone through calcination process, with an average diameter of the powder particle size being 10 µm. This product is produced at a high quality level in terms of high concentration and the smallest particle of calcium hydroxide (Ca(OH)_2_) [12,13,21]. In general, the properties of Ca(OH)_2_ were similar to slaked lime (SL) that is made from limestone, which is an inorganic compound with the chemical formula Ca(OH)_2_. Table-6 shows the properties and ingredients of FdCa(OH)_2_, including standard SL; FdCa(OH)_2_ is a colorless crystal or white powder, and the main ingredient of Ca(OH)_2_ and SL, as 97.06% and 95%, respectively. Both FdCa(OH)_2_ and SL have high alkalinity (~pH12.5). The present study indicated that FdCa(OH)_2_ powder could inactivate NDV in the absence and presence of organic materials, which harmonized with several researchers such as Thammakarn *et al*. [10], who reported that alkaline agents such as scallop-shell powder and SL have high alkalinity and excellent ability to inactivate viruses such as AIV. Lorcharoenrungroj *et al*. [22] and Paditporn *et al*. [16] also reported alkaline agents, such as fresh charcoal ash and SL, that could inactivate bacteria and viruses. In addition, the SL is effects toward consumers and animals such as irritation of skin, eyes, and respiratory inflammation. However, FdCa(OH)_2_ induces these clinical signs less than SL.

**Table 6:** Properties and ingredients of FdCa (OH)_2_ powder.

FdCa (OH)_2_	Properties		
Appearance	White or ashy white color powder and granules		
pH	12.4 (25°C saturated solution)		
Specific gravity	2.24		
Solubility	Dissolves in water Saturated solution forms as 0.17 g/100 cc		
Resolution temperature	580°C		
Acute toxicity	Outside based on LD_50_: 7340 mg/kg within the rat Skin corrosive and circumstantial inflammation Severe damage and stimulate toward eyes		

**Ingredients**	**Chemical formula**	**Ingredient**	**Standard slaked lime**

Calcium hydroxide	Ca (OH)_2_	97.06%	>95.0%
Carbonate object		Clear	Non-bubble
Hydrochloric acid insoluble		Clear 0.01%	<0.50%
Heavy metals		Clear	<40 μg/g
Alkali metal and magnesium	Mg	Clear 0.56%	<6.0%
Barium	Ba	Clear	<0.03%
Arsenic	As_2_O_3_	Clear 0.08	<4 μg/g

FdCa (OH)_2_=Food additive grade calcium hydroxide

In general, the SL or calcium hydroxide could dissolve in water and dissociate into Ca^++^, and OH^-^, resulting in a solution with high alkalinity. This solution has a pH of greater than 9 due to the (OH)^-^ and this is postulated to be the main mechanism of antimicrobial activity against bacteria and viruses [23]. However, the solubility of FdCa(OH)_2_ is lower than SL, as 0.17% and 0.185%, respectively. The present study revealed that both 0.17% and 3% of FdCa(OH)_2_ solution in the absence or presence of organic materials, did not show inactivation differences. These results confirmed that 0.17% represents a saturated solution, which is sufficient for bactericidal and virucidal activities.

Normally, the survival of various viruses, especially AIV, is affected by different physical and chemical conditions, such as temperature, pH, UV, detergent, and salinity [24-26]. These viruses can survive for several months to a year in the optimal environment, especially in water [1], including at low environmental temperatures [27]. Several viruses are stable at a slightly basic pH (7.4-8.2) such as AIV [24]. However, viruses are considered to be sensitive to acidic or high alkaline conditions, including low-pathogenic and highly-pathogenic AIVs [25-29]. The high pH of FdCa(OH)_2_ in the present study also revealed that FdCa(OH)_2_ could inactivate bacteria and viruses even at high pH condition and could not inactivate due to neutralizing by Tris-HCl.

Finally, under harsh conditions, the FdCa(OH)_2_ powder could inactivate both viruses. This experiment simulates real environmental conditions such as under sunlight condition and provides factors of heat, ultraviolet radiation, and dryness. Similarly, emulating summer season, including, wet and dry conditions, was conducted by soaking in water and then drying thoroughly, which resembles rain soaking in the rainy season. These conditions are very important for disinfectant applying in and around animal farms, especially the frequency of using in each season.

## Conclusion

The present study showed that FdCa(OH)_2_, either in powder and solution forms, has good antimicrobial properties against SI, *E. coli*, NDV, and AIV when used at the right dosage and exposure time. The FdCa(OH)_2_ is not only efficacious regardless of the presence or absence of organic materials but it is also stable under the harsh conditions simulated in this study. Finally, the FdCa(OH)_2_ is safe for consumers because it is of food additive grade and can be useful as an alternative disinfectant, especially for biosecurity enhancement in and around poultry farms.

## Authors’ Contributions

SR and KP carried out the main research works and analyzed the main data in the experiments. DP, TJ, and KT have supervised the laboratory work and approved the final version of the manuscript. All authors read and approved the final manuscript.
